# Test procedure for the evaluation of partially automated driving HMI including driver monitoring systems in driving simulation

**DOI:** 10.1016/j.mex.2024.102573

**Published:** 2024-01-16

**Authors:** Nadja Schömig, Christina Kremer, Sebastian Gary, Yannick Forster, Frederik Naujoks, Andreas Keinath, Alexandra Neukum

**Affiliations:** a WIVW (Wuerzburg Institute for Traffic Sciences) GmbH, Germany; b BMW Group (Bayerische Motorenwerke), Germany

**Keywords:** Partially automated driving, Level 2 automation, Human-machine interface, Driver monitoring system, Evaluation, Driving simulation, L2 HMI and DMS testing procedure

## Abstract

The proposed test procedure presents an approach for the evaluation of the usability of partial automated driving HMI including driver monitoring systems in driving simulation. This procedure is based on a definition of requirements that a Level 2 HMI and its included driver monitoring system must fulfill in order to guarantee that the drivers understand their responsibilities of continuously monitoring the driving environment and the status of the partial automated driving system. These requirements are used to define the evaluation criteria that have to be validated in the test as well as the use cases in which these criteria can be assessed. The result is a detailed and comprehensive test guide including the specification of the test drives, the necessary instructions, the test environment and the recruiting criteria for the test sample.

• Evaluation of usability aspects of level 2 automated driving HMI including driver monitoring systems
• Based on the definition of requirements for L2 HMI
• Test guide including the definition of use cases, evaluation criteria and testing conditions in driving simulation

## Graphical abstract

**Figure:**
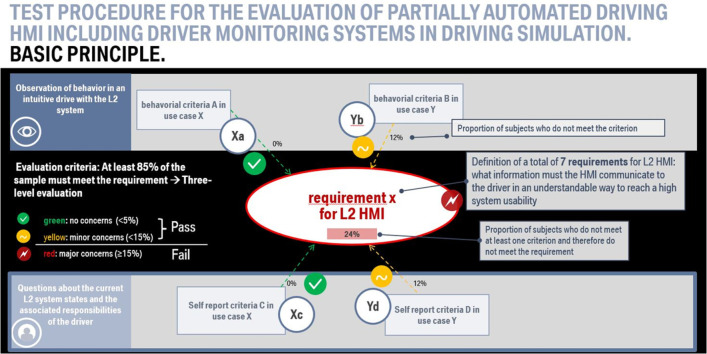
Test procedure for the evaluation of partially automated driving HMI including driver monitoring systems in driving simulation.

Specifications table

**Table:** 

Subject area:	Engineering
More specific subject area:	Human Factors, automated driving
Name of your method:	L2 HMI and DMS testing procedure
Name and reference of original method:	Naujoks, F., Hergeth, S., Wiedemann, K., Schömig, N., Forster Y. & Keinath, A. (2019). Test procedure for evaluating the human–machine interface of vehicles with automated driving systems, Traffic Injury Prevention, 20(1), pp.146-151. https://doi.org/10.1080/15389588.2019.1603374
Resource availability:	Not applicable

## Method details

### Goal and overview of the method

When driving with a partially automated system (Level 2 L2; Society of Automotive Engineers International (SAE), 2021) the driver is still responsible to monitor the roadway and to be ready to react to a system limit or error at any time. In this context, the design of the Human-Machine Interface (HMI) of the L2 system plays a central role for an adequate system understanding. It should adequately communicate the functionalities and limits of the system, e.g. by clearly naming it as “assistance”. In addition, the driver should be able to easily recognize these responsibilities, e.g. by using clear symbols, notifications, text etc. In case the driver forgets or neglect these responsibilities a so-called Driver Monitoring System (DMS) is aimed to detect this and provides means to remember and warn the driver on his/her reduced attentional state and their responsibility to supervise the system.

In order to evaluate whether the HMI of an investigated L2 system with its different modes, functionalities and DMS is effective in fulfilling the requirements of L2 systems to communicate central responsibilities to the driver it is necessary to have one common and standardized test protocol.

Such a protocol is presented in this paper. It focusses on the assessment of the usability aspects of L2 HMIs including the DMS messages displayed in the HMI. This considers, in particular, testing the comprehensibility of the messages, which can be captured via self-reports (answers on specific knowledge questions about the HMI) as well as from the resulting appropriate behavior when using the system. In order to ensure that all requirements an L2 system must fulfil are tested, a requirements catalogue was first created. From that the use cases to be tested and the relevant evaluation criteria were derived. The procedure for developing the test protocol is described in detail below. The test protocol is intended to provide the possibility to examine different variants of L2 systems either by themselves in an absolute sense or in comparison with each other. Moreover, the test procedure is developed with the purpose to get valuable results about the understandability of the HMI at an early stage of development, when only a prototype is available that can be investigated in a simulator study. The method describes a standardized procedure how an investigator (human factors expert, designer, developer) can uncover basic comprehension problems in the development phase of a system. The problems uncovered should then be used to optimize the HMI so that it better adheres to these requirements. The test procedure is explicitly not intended to assess safety aspects. For this purpose, it would be recommended to use other test procedures later in the development process.

The method described here is innovative in the sense that it describes a standardized, use case driven, requirement-based method to evaluate the intuitive understandability of L2 HMI and DMS by a user study in the driving simulator based on absolute self-report and behavioural criteria at an early level within the system development process.

### Definition of requirements for L2 systems

In a first step of the test development process the requirements for L2 systems were defined. In general, a requirement regarding the HMI should be understood as what information the HMI must communicate to the driver in an understandable way to reach a high system usability.

Various premises were agreed upon to derive the requirements for the L2 system: The requirements should primarily address the usability of the L2 system with DMS in the sense of whether a driver understands the outputs of the system and the associated DMS. Aspects of safety of use, such as controllability or response to system errors, are not examined. A use-case related approach is chosen to test the requirements, i.e., the driver's understanding is captured directly in the interaction with the system in a specific scenario and not as knowledge about the system, such as described in a manual. The requirements should also not contain any concrete design specifications (e.g., how exactly the information should be presented). Technical system specifications (e.g., time thresholds of inattention after which warnings are issued) are also not specified.

The following sources were used to derive the requirements for an L2 system. First, it was examined to which extent the five minimum requirements defined by NHTSA for automated driving (ADS systems, L3 and higher; [19]) can be also applied to L2 systems. Following NHTSA voluntary guidance, “at minimum an ADS should be capable of informing the human operator or occupant through various indicators that the ADS is: (1) functioning properly; (2) currently engaged in ADS mode; (3) currently “unavailable” for use; (4) experiencing a malfunction; and/or (5) requesting control transition from the ADS to the operator” [19], p. 10.

Requirement 1 and 2 are also valid for L2 systems: There should be understandable HMI elements that clearly indicate that the L2 system is currently active and whether it is currently fully functional. Misunderstanding this system state might lead to situations where a driver thinks that the system is currently active, while it might be in standby mode actually. In real-word systems this could be avoided by clearly indicating the status of the system via different icons and colors, e.g. indicating the lateral control by a symbol of a steering wheel luminated in green if active and in grey if it is currently in standby mode.

Requirement 3 “… currently unavailable for automated driving“ is especially crucial for L3 systems as those systems are only available in specific Operational Design Domains (ODDs). One example is geofenced routes where it is guaranteed that the used sensors are able to detect the lane markings with sufficient quality for the automated lateral vehicle control to allow the drivers to take their hands away from the steering wheel. The same is true for L2 hands-off systems: Systems like Super Cruise in the US are only available at interstates, but not in urban environments. In contrast, L2 hands-on systems in principle do not have these restrictions although it is recommended to use them only on certain routes, e. g. on highways. What could happen is that at the moment of system activation one partial function of L2, e.g. lateral vehicle control is in standby because the lane markings are currently not detected. In such cases the system might give a notification in terms of a short explaining text “function currently not available”. In systems with both functions integrated, hands-on and hands-off function, it would be possible that hands-on function is activated in an area outside of the ODD if the driver tries to activate L2 hands-off mode. This information must be adequately communicated to the driver, e.g. by an additional pop-up message indicating “hands-on mode is activated”. Otherwise this could lead to safety-critical situations if the driver takes the hands from the steering wheel although the system is not able to handle lateral control in that condition.

Requirement 4 “… experiencing a malfunction of the [HAV] system” is valid for L2 systems in the sense that during active use of the L2 system there may be situations where limitations in the system functionality occur, e g. short-term unavailability of the lateral vehicle control or other subfunctions of the system. This malfunction might be indicated by a specific optical notification icon plus an explaining text in order to avoid inadequate expectations of the driver about the system's function.

Requirement 5 “requesting control transition from the HAV system to the operator” is only valid in this form for L3 systems where the driver is allowed to take him/herself out of the vehicle control loop until the system requests to take over the driving task again. While driving with L2, the driver is still continuously responsible for the driving task, therefore a take-over request is not required and transitions without a warning are possible. Nevertheless, the driver must understand that there is a potential system degradation, either completely or in parts. Most importantly, the driver must understand the continuously required responsibility for the driving task during the entire drive with the L2 system which includes the monitoring of the system and the driving environment as well as the need to keep hands at the steering wheel in L2 hands-on systems. This can be achieved by continuously showing respective symbols or icons, e.g. an eye for the task of supervising or a symbol of hands holding a wheel. Examples for the various state indicators can be found later in a chapter where the results of a validation study using prototypical L2 HMI concepts are reported.

Another source for the derivation of L2 requirements was the so called “Level 2 Driver Monitoring Principles”, published by the Alliance for Automotive Innovation (AAI, 2021). One part of these principles concerns requirements regarding consumer information. It is requested that “The Level 2 system information, including promotional materials, should reasonably reflect the functionality of the system. This may include (but is not necessarily limited to): any Level 2 specific capabilities or limitations, the responsibility of the driver, the Operational Design Domain, and whether or not the driver's performance of one or more of the driving tasks while the Level 2 system is engaged (within its Operational Design Domain) results in the disengagement of the system” (AAI, 2021; p.1). Furthermore, the document states that “[…] the Level 2 system should, at all times, convey information to the driver on the status of the system such that a driver can reasonably discern whether the Level 2 system is engaged or disengaged.” (AAI, 2021; p.1). Although these requirements only relate to consumer information which might be transferred via a user manual or other information channels, we argue that also the HMI must be able to communicate this information.

With regard to driver monitoring and respective warnings the principles pronounce that “it is important that the driver of a Level 2 vehicle is attentive to the surrounding driving environment at all times”. Secondly, “If a driver monitoring system determines or infers that the driver is not engaged in the driving task, then an initial warning should be issued within a reasonable amount of time from when a system detects the driver is not engaged”. Furthermore, “If the driver does not respond to the initial warning from the driver monitoring system, subsequent warnings should escalate and include, at a minimum, some combination of visual and non-visual (auditory or haptic) alerts.” (AAI, 2021; p.2). Examples for such warnings can be found later in a chapter where the results of a validation study using prototypical L2 HMI concepts are reported.

Based on these sources, the authors’ expertise, and the results of a first validation study in a driving simulator (for results see later chapter of this manuscript) the following set of 7 requirements was derived as the most important ones for an HMI of L2 systems and an integrated DMS system (see Table 1). All requirements should be understood as what information the HMI must communicate to the driver in an understandable way.

**Table 1:** Overview of requirements for L2 systems including DMS.

No.	Requirement	Description	Type
1	Responsibilities	The driver understand that he/she has full responsibility for the driving task in L2 mode (need to monitor system state and traffic situation + need to keep hands on the steering wheel in L2 hands-on).	Primary
]	Full functionality	The driver understands that the system is active and fully functional (and accordingly which subtasks are currently supported).	Secondary
3	Limited functionality	The driver understands that the system is only functional in parts (and accordingly which subtasks are currently not supported).	Secondary
4	System inactivity	The driver understands that the system is inactive (e.g. because the system has switched off completely or in parts).	Secondary
5	Operating inputs	The driver understands which active control inputs in L2 mode have which influence on the functionality of the L2 system (e.g. whether the system is subsequently inactive or still active).	Secondary
6	DMS-warnings	The driver understands the DMS warnings (including the consequences of non-compliance).	Secondary
7	Unavailability	The driver understands that it is (currently) not possible to (fully) activate the L2 system.	Secondary

Requirement 1 - the communication of the driver's responsibilities - is defined as the most important primary requirement, while the other six are defined as secondary requirements.

### Definition of use cases

The use cases for testing an L2 system with DMS are defined as all possible system states or sub-states of an L2 system and transitions within the system (e.g. from one sub-state to another sub-state) and to possible other automation levels below (L0 and L1). These were systematized and listed according to the principle that use cases consist of either driving within system states or transitions between system states.

Continuous driving within an automation level is called “steady state”. Here, a normal steady state is distinguished from a degraded steady state. Normal sub-states of L2 can be, for example, a separate hands-on and/or hands-off driving functionality (assuming that there are systems in which both can be driven dependent from different conditions, e.g. different speed limits or ODDs). System-specific special functions, such as automatic speed adjustment to the prescribed speed limit or momentary override of L2 functionality using the steering wheel or accelerator pedal, can also be sub-states of the system. A degraded system state can result due to both a limitation/constraint of the system or the driver. For example, a degraded system state would be given if a sub-function within an automation level is no longer available without the need for a direct transition to another automation level (e.g. lane marking not detected, automatic speed limit adaptation not fully functional). On the driver's side, a degraded state exists when the driver is no longer adequately performing certain subtasks, e.g., his/her visual or physical monitoring task (e.g., if the driver is not attentive enough or does not have his/her hands on the steering wheel). These are the typical DMS use cases in which the DMS would detect these degraded states and would issue warnings. In these use cases, it has to be checked whether the driver comprehends these warnings and reacts adequately to them.

Transitions between system states are differentiated into driver-initiated or system-initiated and whether they are directed upwards to higher automation levels or downwards to lower automation levels. While transitions to a higher automation level must always be driver-initiated, transitions to a lower automation level can either be initiated by the driver or triggered by the system (system-initiated; due to a functional limit). Transitions from L2 to higher automation levels, in particular to L3, are not considered in the test protocol, since these are out of scope of the present protocol for driver assistance systems exclusively. The ones to automated driving systems are checked in the corresponding test protocol for level 3 systems [20].

This taxonomy leads to a set of use cases, which is listed in Table 2. This list should be understood as an overview of all possible use cases from which a subset has to be selected for the specific system that one wants to test. For example, if a system does not include the two L2 modes hands-on and hands-off but only one, the use cases concerning the transitions between the two modes can be skipped as well as all the use cases related to that mode.

**Table 2:** Overview of all possible use cases with description. A subset may be selected for a specific system to be tested.

Use Case Type	Description	Rationale/what should be experienced
Normal steady system states within L2	L2 hands-on driving	Hands have to be kept on the wheel
	L2 hands-off driving	Hands can be taken off the wheel
	L2 specific functionality- e.g. driving- automatic speed adaptation	Specific additional feature of L2
	L2 driving- driver oversteers	L2 remains active after that action
	L2 driving- driver overaccelerates	L2 remains active after that action
Degraded steady states within L2 (either driver or system-related)	System is (partially) unavailable in L0 (or after activation attempt only one subsystem activates, the other remains in standby).	System enters L2 state, but the lateral control remains in standby
	Lateral control temporarily fails	Lateral control temporarily fails, but the system remains in L2
	Specific system functionality temporarily fails	System functionality fails, so this task must be performed by the driver
	Driver does not fulfil visual monitoring task (looks away from road for too long or too frequent)	DMS functionality becomes active due to non-fulfilment of the monitoring task, resulting in Eyes-Off Warning (EOW)
	Driver does not fulfil physical monitoring task (does not have hands on steering wheel for too long)	DMS functionality becomes active due to non-fulfilment of the monitoring task, resulting in Hands-Off Warning (EOW)
Driver-initiated upwards transitions (either upwards or downwards)	Driver activates L2 from L0	Active L2 Mode after activation and associated tasks/responsibilities (as distinct from L0).
	Driver activates L2 from L1 (ACC)	Active L2 Mode after activation and associated tasks/responsibilities (as distinct from L1).
	Driver deactivates L2 completely (to L0) by braking	System switches off completely due to braking
	Driver switches from L0 to L1 (ACC)	L1 is its own activatable system and not just a sub-stage of L2
System-initiated transitions (within L2 and downwards)	Transition within L2: from hands-on to hands-off	System has two sub-modes and independently switches between them
	Transition within L2: from hands-off to hands-on	System has two sub-modes and independently switches between them
	L2 system deactivates completely	Transition from L2 to L0, in which the driving task must be taken over completely
	Due to system limit or error, only ACC is still active	Transition from L2 to L1, after which lateral control must be taken over
	L2 System deactivates itself by not observing the DMS warnings.	System behavior if there is no reaction at all to the DMS warnings

### Definition of test logic and evaluation criteria

**Fig. 1:**
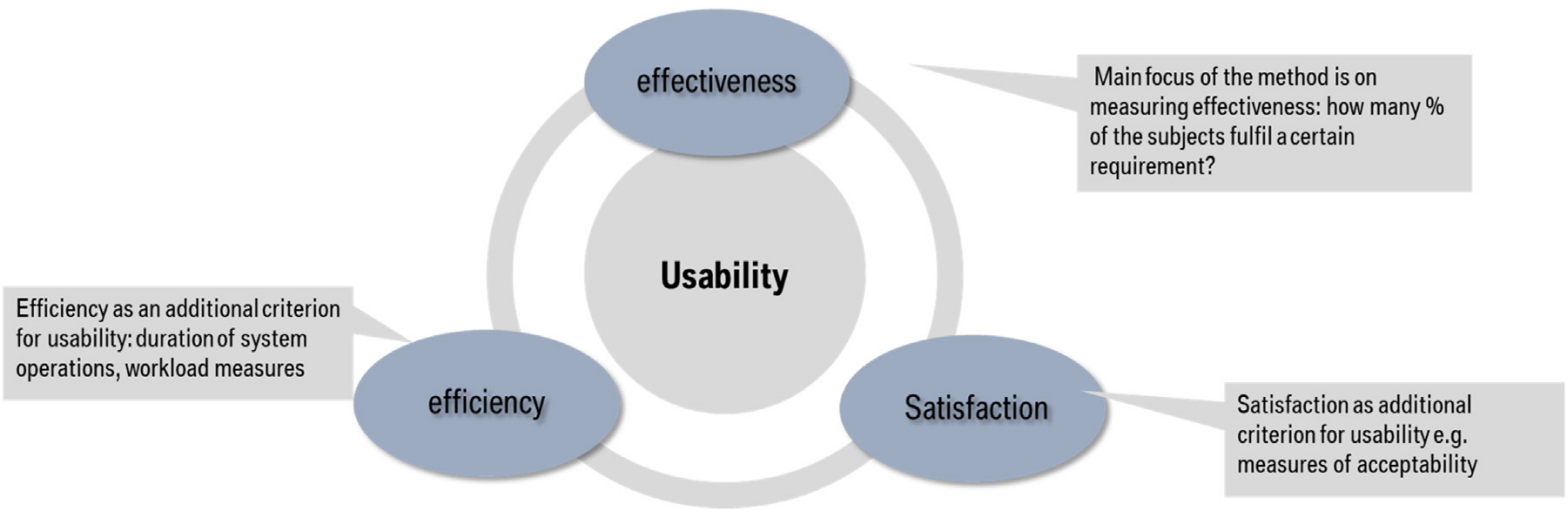
Multidimensional criteria for usability assessment (according to ISO 9241; ISO 2018): Main focus of the test procedure is on the assessment of effectiveness.

The focus of the protocol is to test how effective the HMI is in transferring information to the driver and thereby meeting the requirements of the L2 system HMI and its integrated DMS (as one dimension of the concept usability, see Fig. 1).

The term “effectiveness” refers to the “accuracy and completeness with which users achieve specified goals” (ISO 9241 11:2018, definition 3.1.12). This involves checking whether the drivers can perform a specific task and what errors they make. This check should primarily be carried out during initial contact in order to determine whether a system is intuitively understandable. The simplest measure for this is the task success rate, i.e. what percentage of test subjects in a sample can successfully complete the task. According to Nielsen (1993), this measure is easy to understand and represents the bottom line of usability. It is also interesting to know whether the goal is achieved directly or whether errors are made during operation. In the presented test protocol, effectivity is assessed via an analysis of how many participants in a given use case meet an evaluation criterion (see next chapter for what these are).

The second part of usability is efficiency, which is understood as the “resources used in relation to the results achieved” (ISO 9241 11:2018, definition 3.1.13). Efficiency can be measured using temporal aspects of behavioral measurements (so-called time-on-task measures), i.e. how quickly the driver needs to activate the function, for example. Measures of gaze behavior, such as the number of glances required or the total time spent looking to complete a task, can also be recorded as efficiency measures. In addition, classic workload measures can also be used, where the user can provide information about how much effort he/she had to make to complete a task. To assess efficiency, it is not usually the initial contact that is considered, but the user's subsequent interactions, if it can be assumed that the system is used without errors. As the focus of the presented method is to evaluate the intuitive understandability of the system's HMI this aspect is considered to play a subordinate role.

The third component of usability is satisfaction, which is defined as the “extent to which the user's physical, cognitive and emotional responses that result from the use of a system, product or service meet the user's needs and expectations” (ISO 9241 11:2018, definition 3.1.14). This component is usually recorded at the subjective data level, in which the driver provides information on how satisfied they were with the task processing. Satisfaction measures are almost always recorded in addition to effectiveness and efficiency measures. For the presented method, the recording of satisfaction, i.e., whether drivers like or dislike the HMI is not relevant for the final decision about passing or failing in the test overall. This gets more relevant in the later usage of the system as a whole. Nevertheless, these aspects can be captured during the testing procedure via certain additional measures, such as the System Usability Scale [4].

Effectiveness in the presented method is measured by how often certain predefined criteria defined for a requirement are met. It was decided that this should always be achieved by considering a combination of at least one self-report criterion and one additional behavioral criterion. Ideally, the two measures complement each other.

Self-report measures are on the one hand questions asked directly about the current system state in the situation, which are intended to capture the understanding of the messages and indicators. The comprehensibility of an HMI element should be measurable by the extent to which the driver is able to name clearly what state the system is in, what the system is doing and what his/her responsibilities are. In addition, the participants are required to name a correct indicator of the HMI from which he/she can recognize this. An indicator can be a visual element in the form of an icon or text or an auditory element in the form of a speech message or a warning tone (e.g. in the case of a monitoring request). Asking the driver directly in the situation helps to avoid memory effects that would occur if the driver is asked only after the complete drive.

In addition, the driver should behave correctly and, for example should keep the hands on the steering wheel in a system that explicitly requires this. However, from this behavior alone it cannot be clearly defined, whether the driver is possibly only maintaining the hands on the steering wheel due to uncertainty or lack of system trust and not because he/she knows that this is a current requirement of the system. For this reason, an incorrect behavior should only be evaluated as such if the driver makes respective statements indicating that the cause is actually a lack of understanding. To answer this, surveys after the drive are used, which capture the generated, explicit knowledge about the basic system states of a system, i.e. the mental model. In the end, the self-report criteria therefore achieve a higher explanatory power compared to the behavioral criteria.

The next step in the process of defining evaluation criteria is the definition of thresholds for fulfilling the requirements: For each criterion, the number of participants who do not fulfil it is recorded. If the criterion is recorded in more than one use case, this frequency is determined according to the proportion of subjects who do not fulfil the criterion in at least one of the use cases.

**Fig. 2:**
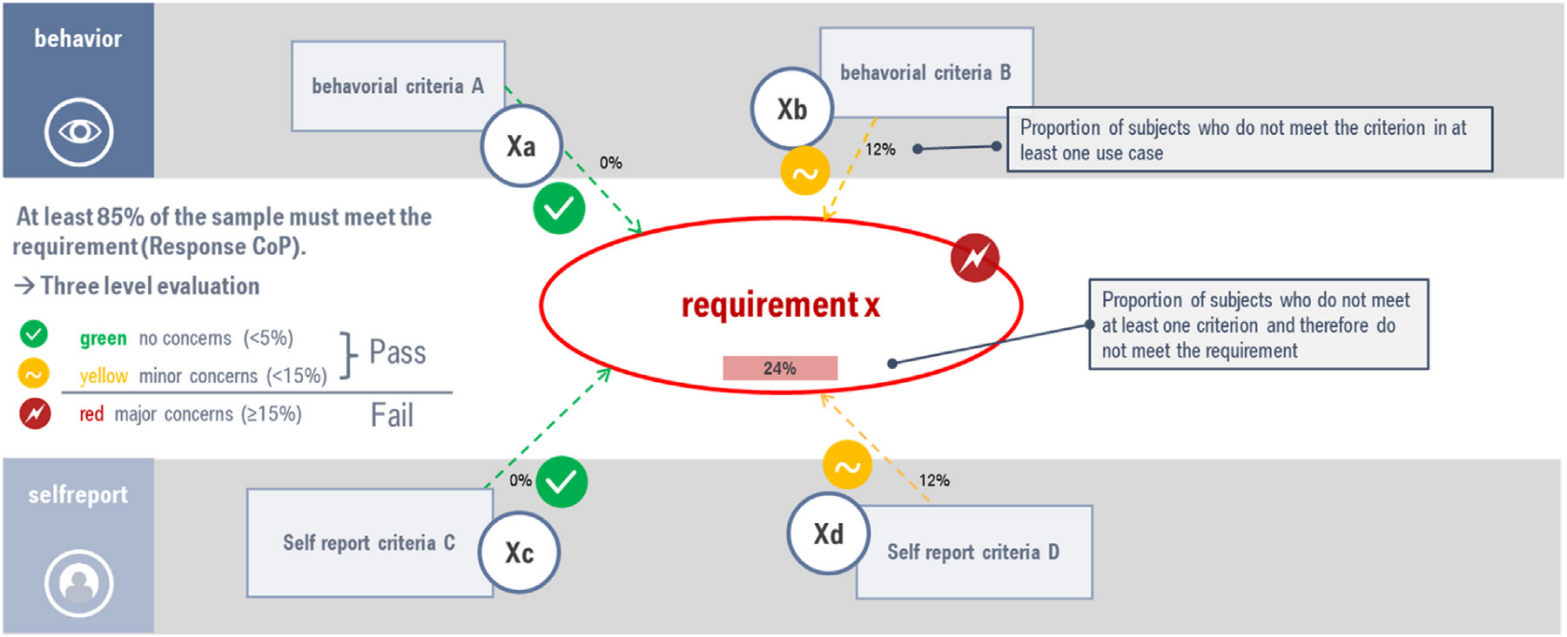
Evaluation principle based on percentage of sample fulfilling behavioral and self-report criteria for a certain requirement x.

From this, the total percentage of the sample that do not meet at least one criterion and therefore do not meet the requirement is calculated for each requirement. The test protocol aims at verifying that 85% of the population will accordingly understand the system and user interface. If the percentage is higher, the requirement is considered not met. Below the 15% limit, the rating is further defined in “no concerns” for a percentage below 5% (green category) and in “minor concerns” for a percentage between 5 and 15% (yellow category). For more information regarding sample size see chapter “Requirements on study sample characteristics”. If an HMI achieves a rating in the yellow or red category, it must be revised in such a way that its comprehensibility is increased. In the case of a rating in the red category, this revision is mandatory, in the yellow category, it is recommended. Fig. 2 illustrates the evaluation principle based on the percentage of sample fulfilling behavioral and self-report criteria for a certain requirement x.

### Definition of test drives

As defined in the previous chapter, both behavioral and self-report measures are intended to serve as criteria for testing compliance. However, it is difficult to collect both types of assessments at the same time if the questions are predominantly intended to capture the person's current state knowledge, and therefore must be asked directly in a situation. These questions during a drive about different system states would strongly influence the subsequent behavior, so that the behavior could no longer be measured intuitively. Therefore, a multi-part procedure composes the test protocol.

In an initial “test drive A”, the focus is on purely observing behaviour (see Table A1). Prior to this drive, the driver receives only rudimentary information about the system being driven (with the aim of knowing how to activate it). The aim is then to capture the subject's direct, intuitive experience of the system as natural as possible. During this drive, the test person experiences the use cases in a natural sequence (which is ensured by implementing plausible driving scenarios) and is not disturbed by additional questioning.

This drive A is followed by an intermediate interview. Here, the participant is asked to describe the system he or she has experienced. This can be used to check which mental model they formed about the system. In addition, the driver can be asked specifically about observed misbehavior that he or she exhibited in drive A to draw conclusions about the accuracy of the mental model. Answers such as “I wanted to try out what happens” lead to the fact that the behavior shown is not included in the evaluation as incorrect. Further optional surveys at this point allow the assessment of additional aspects of usability, acceptance or user experience to be recorded by means of standardized questionnaires.

This intermediate survey is followed by another “test drive B”, which includes in-situation surveys of the current system states (see Table A2). Here, drivers either receive specific instructions to reach a desired target state (here, however, only the activity is instructed, not the state itself, i.e.: “please press the right steering wheel button”- Not: “please activate the system now”) or the experimenter triggers the corresponding system states and transitions via the simulator software (e.g. take-over request). In this drive, no explicit situation needs to be created to make the transition plausible (e.g., a sharp curve that the system's lateral guidance cannot handle). The driver is then asked directly about the system understanding in each use case.

If drivers make erroneous statements after drive A, they are not informed about the correct behavior. It may thus be that they enter the second drive B with false assumptions.

In particular, for the two use cases that investigate whether drivers understand DMS warnings resulting from inadequate driver monitoring, it is necessary to define how drivers should enter these degraded states. In drive A this can be achieved indirectly by instructing drivers to complete a non-driving related task (e.g., search a song in the user interface) or simply observe if this happens on its own. However, it can happen, that the driver may then not initially experience a DMS warning. At least in the second drive B, the drivers should be directly asked to take their hands off the steering wheel/eyes off the road for a specific activity in order to question them about the DMS warnings triggered by this.

### Definition of self-report measures and observed behavior in the defined use cases

In order to check whether the total of 7 defined requirements are met, different self-reporting and behavioral criteria were subsequently defined for each requirement. This includes the specific indicators, i.e., measures of how these must be recorded in the different UCs. In general, at least one self-report measure should be defined as “minimal criterion” which is needed at minimum to fulfill an entire requirement.

By way of example, Table 3 shows the criteria for requirement 1: The driver understands that he/she has full responsibility for the driving task in L2 mode. For this requirement, one behavioral criterion and three self-report criteria were determined. The behavioral criterion is defined as whether the driver receives eyes-off warnings from the DMS systems or whether he/she removes the hands on the steering wheel in the use cases in which hands-on driving is required. If a misbehavior is observed, the driver is asked in the interview after drive A why he/she did this. Only if the answer suggests that an incorrect mental model is the cause (e.g., “Answer: I thought I was allowed to take them away”), the misbehavior is counted as such.

**Table 3:** Evaluation criteria for requirement 1: “The driver understands that he/she has full responsibility for the driving task in L2 mode”.

Criteria type	Criterion	Indicator/measure	Evaluation	Use Case where indicator is assessed
Behavior	a) Driver adequately monitors the traffic situation and fulfils his/her responsibility	Observation of whether driver triggers hands-off or eyes-off warnings due to erratic behavior If yes, question in interview: “Why did you take your eyes off the road?” or “Why did you take your hands off the steering wheel?”	Score as incorrect only if it is clear in interview that incorrect behavior is caused by an incorrect mental model	Drive A: For EOW: all UC for HOW: all L2 hands-on UC with necessity to keep hands at the wheel L0/L1_L2_activation, L2_HandsOn_1/2, L2_HandsOn-SetSpeed_1/2, L2_HandsOff_On
Self-report	b) Driver has explicit knowledge about responsibility (minimal criteria to be fulfilled)	Question in interview: Which system/systems did you just drive with (which system did you just activate by the xy key?)- Default SAE description of L1, L2, and L3.	Correct answer: L2	Interview after drive A
	c) Driver has explicit knowledge of responsibility to monitor traffic events	Question in drive B: Do you need to monitor the traffic situation?" By which system outputs do you recognize this?	Correct answer: “yes” and naming of a correct HMI output	Drive B: L2_HandsOn & L2- HandsOff
	d) Driver has explicit knowledge of responsibility to keep hands on steering wheel in L2 hands-on	Question in drive B: Do you need to keep your hands on the steering wheel? By which system outputs do you recognize this?	Correct answer: “yes” and naming of a correct HMI output	Drive B: L2_HandsOn

One specific question in the interview after drive A is considered as a self-report measure: The driver is supposed to describe the system currently driven as a Level 2 system with its function, subtasks and responsibilities (see chapter “Description of Test Protocol” for the asked question and answer categories). Additional self-report measures are collected in drive B. Here, the drivers are specifically brought into the L2 hands-on state and are asked to answer the question “Do you need to keep your hands on the steering wheel now?” With “Yes”. Regarding the question “By which system outputs do you recognize this?” they should name a correct HMI element (e.g. a symbol in the cluster display showing hands on the steering wheel or similar). In addition, they are asked both once in a hands-on condition and once in a hands-off condition, “Do you need to monitor the traffic situation?” (correct answer: “Yes”) and “By which system outputs do you recognize this?” (naming of a correct HMI output, e.g. an icon in the cluster display showing an eye or similar). For this requirement 1, the minimal self-report criterion could be criterion 1b) that the driver is able to name the used system as L2 system together with the related responsibilities in the interview after drive A.

### Example for the evaluation principle for requirement 1

**Fig. 3:**
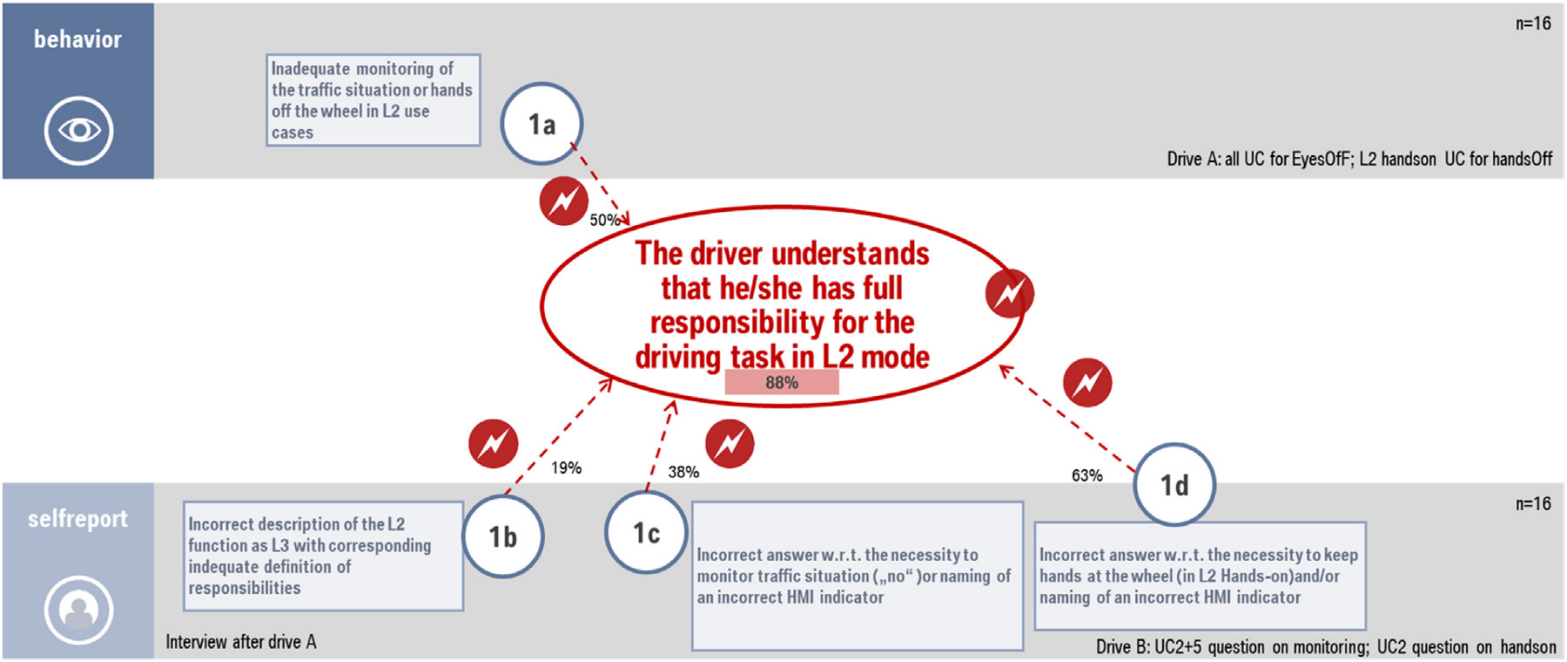
Example for the evaluation of requirement 1 for the reduced HMI prototype investigated in the validation study.

In order to get an impression how the evaluation process will be conducted an example is given here from the validation study described in the later chapter “results of a validation study” (see Fig. 3). During the conduction of the study it turned out that half of drivers experiencing the reduced HMI design prototype received hands-off warnings due to the fact that they did not know that they need to keep their hands on the steering wheel (criterion 1a). 10 of the 16 drivers in this group said in drive B they can take hands-off in L2 hands-on mode (criteria 1d). 3 drivers already argued in the interim interview that they believe the “PLUS” (intended to indicate the hands-off mode) means they are allowed to do side tasks (criterion 1b). 6 drivers said in drive B that they don't have to supervise in L2 hands-off mode (due to the “PLUS”; criterion 1c). Altogether this results in 88% of the sample that hurts at least one of the criteria. Therefore, the requirement has to be defined as not fulfilled with mayor concerns.

### Definition of instructions

In order to make the first drive A as intuitive as possible, the participants only receive reduced information about the system. However, in order to give the driver an indication of the type of system and what to expect in principle, the test person receives the information that in principle there can be three different systems in the vehicle (meaning L1, L2, L3). The basic functionalities of these three systems are described to the driver:

• L1: system that provides longitudinal guidance,
• L2: system that provides both lateral and longitudinal guidance. For both L1 and L2 systems, the driver must continue to monitor the driving task.
• L3: System that takes over both lateral and longitudinal guidance and, in addition, offers the possibility of engaging oneself in other activities, since in L3 the takeover request is given some time before the system limit.

In the instruction, the terms “assistance” (for L1 and L2) and “conditionally automated” are used for L3. Special functionalities of automation like e.g. an automatic speed adjustment are explicitly not mentioned as well as possible sub-modes of L2 (hands-on vs. hands-off).

Furthermore, it is not mentioned that the system has an integrated driver monitoring system and this is not described accordingly. To ensure that the participants had understood the various systems and their functionalities, they are asked to explain the individual systems in own words after the instruction, including the subtasks and responsibilities assumed. If it becomes apparent that they have not understood aspects, the instruction is repeated accordingly. In order to be able to operate the system, the controls for activating/deactivating the systems are shown or described to the driver (e.g. by naming the button as “the one in the middle at the right side of the steering wheel”).

### Description of test protocol

In order to guarantee a standardized testing procedure, the experimenter should use a test protocol which contains all the relevant information needed to conduct the study and to assess all the relevant self-report and behavioral indicators. This includes instructions before and during the drives, the sequence of the conducted use cases per drive, the required inputs into the simulation software for triggering certain system states and their timings, the questions to ask and behavioral indicators to observe during the use cases.

For each use case in drive A it is recommended to describe the start and end point on the route and behavioral categories the experimenter can use to directly classify the observed behavior as correct or incorrect. If the experimenter must trigger certain system states, the time point for this task and the respective action should be described in that use case. Additionally, the test protocol should allow the experimenter to write down some additional remarks or comments by the driver.

The interview protocol after drive A should contain a sheet where experimenter can note the answers of the subjects with regard to the question which system they were just driving with. The elements named by the subject should be classified into the ones that are necessary to describe the system correctly as L2 (esp. responsibility for monitoring should be named) or incorrectly as L1 (only longitudinal control is performed by the system) or L3 (e.g. if drivers explicitly state that they could direct their attention towards other non-driving related activities). In addition, predefined questions about the reason for inadequate behavior in drive A should be included in the interview sheet. The answers given by the subjects should be protocolled in a way that it gets clear whether the driver showed this behavior due to an incorrect mental model or due to some other irrelevant reasons. Here, memory effects might occur when the subjects incorrectly remembered the situation where they showed this behaviour or when they are not able to remember that situation at all. In such cases the experimenter tries to ask more specifically. In case the answer still does not clearly hint to an incorrect mental model, the behavior should not be defined as inadequate.

For each use case in drive B it is recommended to describe the actions the experimenter has to do to bring the system into the required start state for this use case (either the instruction what the subject is told to do, e.g. press button XY, or the how the state should be trigged via the simulation software). Furthermore, the use case should contain the sequence of questions that have to be asked together with predefined answer categories that allow to directly classify the answers as correct or false. The protocol should also allow to write down alternative not foreseen answers. In the best case, the test protocol is designed in a way that the relevant self-report and behavioral can directly be coded as correct or false.

### Recommended adaptations of the test protocol

Depending on the specific system and HMI to be tested there might be a need for several adaptations of the test protocol. This might refer to the inclusion, replacement or deletion of use cases according to the investigated system. For example, it may be that a system does not include specific features like automatic speed adaptation or it includes either hands-on or hands-off functionality but not both. If the integrated DMS only includes either hands-off or eyes-off detection the respective other use case can be omitted.

In addition, the behavioral categories in drive A might be adapted to specific system characteristics, e.g. if the driver is requested to adapt speed for him/herself the correct behavior in the respective use case is “timely speed adaptation after speed limit change”. For drive B an adaptation of the answer categories is requested, e.g. according to specific HMI characteristics of the L2 system and the outputs that are used to display the system states and transitions which have to be named on the question: “By which system outputs do you recognize the current system state?”.

Therefore, the method requires an intensive preparation of the necessary experimental materials as well as a good familiarization of the experimenters with the experimental protocol.

Further adjustments may be recommended based on the focus of the study: If the aim is the overall validation of the L2 system as presented here, the methodology should test all seven requirements and use all use cases from the defined use case set accordingly (including normal functionality, restricted functionality, all transitions, and the DMS). The results of a simulator study using this methodology will be described in a later chapter in this publication and, more detailed, in a further publication by Schömig et al. (manuscript in preparation).

Moreover, it might also be that the focus is primarily on safeguarding/evaluating the DMS. Then it is sufficient to test requirements 1 ("The driver understands that she/she has full responsibility for the driving task in L2 mode") and 6 ("The driver understands the DMS warnings including the consequences of non-compliance") by a reduced use case set consisting of the use cases with normal functionality and the DMS use cases.

Moreover, the requirements defined should be weighed against each other in the evaluation. The current proposal is that requirement 1 is the most important one, as it is of highest relevance that the drivers do not mix up the L2 system with an L3 system where full responsibility moves over to the system with the potential safety-critical consequence that the traffic environment is no longer observed.

Another point of discussion is the question of how many evaluation criteria should apply as the basis for fulfilling a requirement and how they are weighed against each other. Currently, the proposal is to define at least one self-report criterion as so-called “minimal criterion” for each requirement which is needed at minimum to fulfill an entire requirement. Behavioral criteria observed in the intuitive drive might be added to the minimal criteria but should be only interpreted as problematic if the erroneous behavior is tied to a wrong mental model and not simply due to exploration of the interface and the system.

## Other methodological issues

Requirements on study sample characteristics: With the background that absolute criteria will decide about the acceptance/non-acceptance of a tested HMI the minimum requested sample size to derive this statement with a statistical probability must be defined. In order to state with a statistical probability of 5% that this event does occur in the population with a probability of less than 15% a minimum number of 20 subjects is required. This number is therefore proposed in the Response Code of Practice [21] which describes test requirements for controllability assessments of assistance systems: a sample size of 20 subjects is recommended which all have to pass a certain criterion in order to suppose controllability for 85% of the population [21]. The same number is stated as necessary for the analysis of quantitative data in usability tests with the aim to reach a preferably low confidence interval (see [18]). From these rationales the recommendation for the sample size is derived: For the evaluation of the fulfilment of the requirements it is recommended to examine at least 20 subjects plus additional over-recruitment, so that finally at least 20 valid data set per HMI or DMS condition are available (one of the prerequisites for a data set to be considered valid is that a subject understood the instruction). For a more detailed overview on sample sizes in validation and verification studies and potential number of participants that may fail a criterion (but where the protocol still allows a signoff) see Forster et al., [8].

With regard to the age distribution of the sample there are few recommendations given in the methodological literature. However, it can be expected that age might have an influence on the studied topic in the way how much workload drivers experience in interacting with an L2 system (Molnar et. al., 2017) and how much they trust such systems [7]. As differences can be expected and as all age groups might use such automated systems it seems meaningful to select a heterogeneous sample with regard to age including young-aged, middle-aged and older-aged drivers. According to the NHTSA distraction guidelines [17], the sample should be evenly distributed among four age groups, oriented to the U.S. population: equal numbers of subjects from each of the age groups: 18-24; 25-39;40-54;55 and older. Since no gender effects are expected, it is recommended to base this sample property on the gender distribution of approximately 50:50 in the population.

With regard to the question of how much prior experience with assistance systems and automated systems the sample should have, the test protocol recommended here is again based on the Response Code of Practice [21]: According to this, naïve subjects should be tested in relevant scenarios. Naïve means that the subjects have no more experience or prior knowledge of the system than a later customer. However, this requirement will become increasingly difficult to fulfil in the future as L1 and also L2 systems continue to penetrate the market. Under these circumstances, the requirement should be that at least all drivers have the same degree of experience with such systems and do not differ too much in their prior experience.

Requirements on driving simulation: The driving simulator used for the study should fulfil the following requirements: Regarding the L2 functionality, the implemented system must be able to automatically perform the longitudinal and lateral vehicle control on the basis auf simulated sensors. The DMS system must be implemented according to the real system (e.g. including hands-on sensors in the steering wheel and/or a camera system allowing eye tracking). Since the automated system and DMS are tested under laboratory conditions, no further assumptions about reliability of these technological features are made. Thus, the present approach suggests to implement a DMS with cameras that support a highly reliable tracking of the participants in the study. The system logic should be based on the state diagram of the real system including all defined transitions between the system states (together with the necessary operations and conditions). The HMI must include all specified outputs, e.g. visual (e.g. cluster display, HUD, LED), auditory (sounds, speech outputs) and haptic elements (e.g. seat vibrations).

The driving simulator itself can be static as well as dynamic. It must allow the observation of the driver and the driving environment in order to observe all the defined behavioral criteria. In best cases, displays show one view on the driving environment (birds eye view is favored), one view to the face of the driver and one view on the HMI elements (operation devices, visual displays) and the position of driver's hands.

## Results of a validation study

A driving simulator study was conducted to validate the test methodology described. It used two exemplary, prototypical HMI variants of an L2 system. It was examined whether the method presented can be used to identify differences with regard to the degree of fulfilment of the seven requirements.

**Fig. 4:**
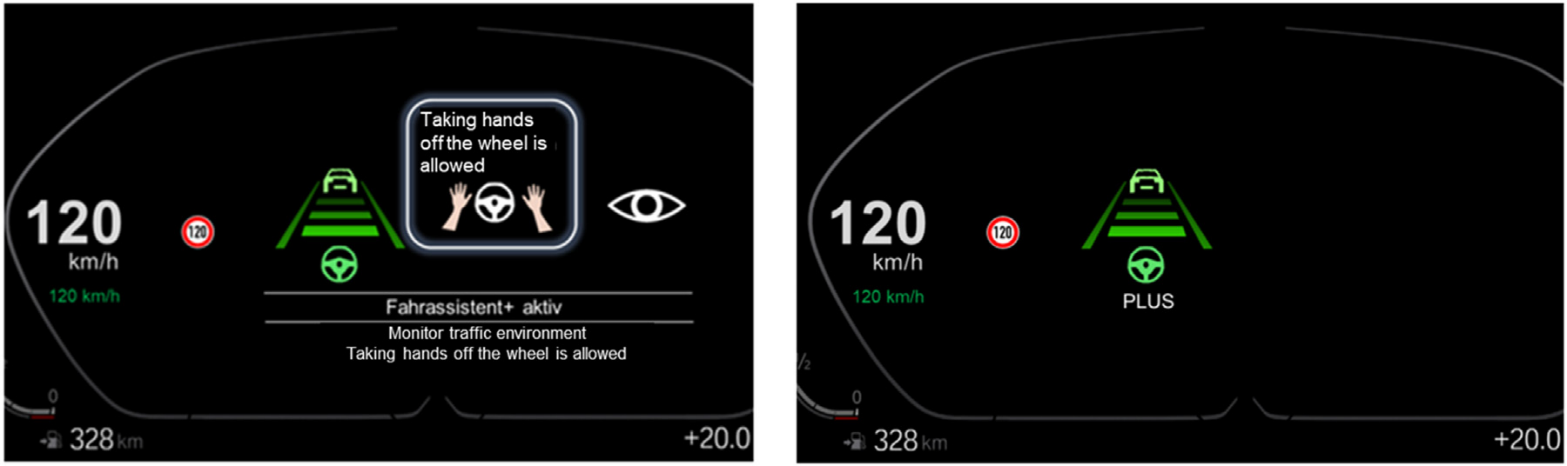
Exemplary HMI status for the transition to hands-off driving mode in the enriched HMI version and the reduced HMI version (translated in English; original graphics used in the study were in German language).

**Method:** The study was conducted in the static driving simulator of WIVW using the driving simulation software SILAB®. The L2 HMI was displayed in the instrument cluster display behind the wheel. Two different HMI design prototypes were compared: one enriched HMI including many redundant information about system states and transitions and one reduced HMI including only basic information about the system. Both systems showed the basic system state by means of a visual icon showing a “road” with lane markings and a steering wheel symbol. While the redundant HMI showed only this information, the enriched HMI contained several additional features: a text display with the system name ("driving assistance" for L2 hands-on functionality or "driving assistance +" for L2 hands-off functionality) and the naming of responsibilities (monitor and hands-on/hands-off) and additional icons for hands-on responsibility and for monitoring task. Furthermore, transitions between system states and DMS warnings were accompanied by speech outputs, additional sounds and clear pop-ups (see Fig. 4 as an example for the transition to hands-off driving mode).

The included Driver Monitoring System (DMS) consisted of an eyes-off detection and a hands-off detection. The hands-on detection worked by a capacitive steering wheel. The eyes-on detection worked with the eye tracking system from SmartEye. All glances away from the forward road are defined as distracted including glances towards the instrument cluster (where the L2 HMI was presented). Hands-off warnings were indicated using the steering wheel icon, eyes-off warning using the eye symbol. Depending on the warning stage they were illuminated in yellow and red and combined with an acoustic feedback depending on the warning stage (first warning stage: after 15 s hands-off/after 4 s eyes-off; second warning stage: after 30 s hands-off/7 s eyes-off, third warning stage (system deactivation): after 1 minute hands-off/10 s eyes-off).

For the study, the above described test methodology was used with the described use cases. *N* = 32 participants experienced the L2 system in the study, *n* = 16 with the reduced HMI and *n* = 16 with the enriched HMI (less than the recommended 20 subjects per group). 11 participants were female, 21 were male. Mean age of the sample was 43 years (SD = 17.7 years).

Summary of results: The data analysis concentrated on the evaluation of the fulfilment of the seven requirements for the two HMI conditions. Separately for each requirement, the defined behavioural criteria as well as the self-report criteria were recorded in the respective use cases and evaluated with regard to the percentage of drivers who did not fulfil the criteria. With regard to the evaluation criteria described above, requirements were evaluated based on the proportion of subjects in the corresponding condition who did not meet at least one criterion: no concerns if less than 5% of the sample teared the requirement (1 out of the 16 subjects per group), minor concerns if less than 15% teared the requirement (3 of the 16 subjects) and major concerns if the value raised above 15% (more than three subjects).

In Table 4 the percentages and the evaluation categories are shown. It got obvious that there was a systematic lack of understanding of the hands-on requirement in L2 hands-on mode in the reduced HMI (requirement 1). Some drivers thought that hands do not have to be taken to the steering wheel at all or only for a short time. Furthermore, L2 hands-off was partially interpreted as L3 where the engagement in a non-driving related task is allowed and the driver does not have to monitor any more.

**Table 4:** Summary of HMI evaluation (no, minor, mayor concerns) per requirement and their fulfilment rates.

The negative evaluation of requirement 2 in both HMI conditions resulted from the fact, that regardless of the HMI variant, some test subjects permanently overrode the system via the accelerator pedal and never tried out what happened when they released the gas pedal and subsequently misinterpreted the displayed system state is the one showing full functionality. Furthermore, in the reduced HMI, L2 was partly described as a system in which the hands can stay away and only need to be taken to the steering wheel for a short time; the missing PLUS icon was sometimes interpreted as the system not being fully functional.

All drivers reacted adequately if the system went into a state with limited functionality (requirement 3) - only one driver did not notice the transition. In general, there was a basic understanding of limited functionality – although many drivers named the currently active system mode as ACC (and not as L2 which was actually still active). As a consequence, they named this system (ACC) as “functioning properly”. Regarding the system state inactive (requirement 4) no systematic lack of understanding existed: only in individual cases the wrong system indicator was named or the transition to L1 was not perceived. Regarding the consequences of system operating inputs (requirement 5) there was no systematic misunderstanding: only one participant waited to see whether the system after deactivation accelerated itself again.

What could be observed was a systematic lack of understanding of the hands-off warning (first part of requirement 6) for some participants in the reduced HMI (with altogether more frequent warnings): this was due to a partial wrong attribution of the reason for the hands-off warning and an inadequate assumption that only a short-term reaction to the hands-on warning is required. In contrast, the eyes-off warnings (second part of requirement 6) were understood almost very well, despite one participant of each condition which did not understand the eye-symbol. With regard to requirement 7 there was no problem at all.

The detailed results of the validation study will be reported in Schömig et al. (manuscript in preparation).

## Limitations

One limitation of the method is that, as already described, it only tests usability aspects of the HMI. Full system validation, including safety of use (safe use of the system within the system limits) and controllability (safe control of the system at system limits and in the event of system errors), must be performed via further tests (see for example [13] for safety in use aspect of mode awareness). Alternatively, the presented test procedure could be repeated in a later stage of the development process, then including more diverse scenarios that test the system's response and the respective drivers’ reaction under various driving conditions and potential hazards either in a driving simulator environment but even better in a real vehicle, e.g. on a test track.

In addition, within the scope of user testing in a driving simulator session of approximately two hours, statements can only be made about the user's initial, intuitive interaction with the system. The development of system understanding over a longer period of time or even possible negative processes, such as behavioral adaptations or system misuse with increasing and longer system use cannot be considered here.

At the moment there is no regulatory binding framework for empirical validation of HMIs of vehicles equipped with L2 automation and additional DMS. Thus, the present approach does not represent a binding test framework for signoff against legally binding criteria. It outlines a best practice recommendation for human factors experts to systematically test L2 HMIs against state-of-the-art criteria that were identified beforehand.

## Ethics statements

Every study conducted at the WIVW follows a regulated ethical review procedure. The ethical guidelines and principles are based on the Guidelines for Safeguarding Good Research Practice of the German Research Foundation (Deutsche Forschungsgemeinschaft, DFG) as well as the Code of Professional Ethics of the German Association of Psychologists (Berufsverband Deutscher Psychologinnen und Psychologen e.V., bdp) and the German Psychological Society (Deutsche Gesellschaft für Psychologie e.V., DGPs). This includes that the relevant informed consent was obtained from all subjects that had participated in the validation process of the method. Furthermore, every study that raises ethical questions is reviewed by an ethics advisor by means of an internal ethical review process. The advisor checks the ethical acceptability of the study objectives and procedures and, if necessary, requests measures to ensure that the study is conducted in an ethical way. The study will not start until it has been judged to be conducted in an ethical and safe manner. All employees involved in the studies are regularly trained in the ethical principles and procedures of the WIVW.

## Additional information

### Rationale: State of the art of the evaluation of HMI and DMS for L2 systems

For the European market until 2022, it was required for all L2 systems that the driver keeps the hands at the steering wheel. Regulation R79 [25] makes clear statements about how a hands-on detection system must react in case the driver does not fulfil this responsibility and how to warn in a specified escalation sequence to put the hands back to the wheel (optical warning after 15 seconds, optical and acoustic or haptic warning after 15 additional seconds, switching off the automation after 30 more seconds).

In contrast, so called hands-off systems that allow the driver to take away the hands from the steering wheel have been permitted and are on the market in the United States for some time now (e.g. Super Cruise available in several Cadillac models, Blue Cruise available in Ford Mustang, Extended Traffic Jam Assist). In 2023, BMW received the approval of a Level 2 hands-off driver assistance system (Assisted Driving Plus) also for the German market [27]. Within a German project financed by the VDA (German Association of the Automotive Industry; VDA report 216300; [12]) several studies on hands-off driving functions were conducted and compared with hands-on functions (user surveys, Field Operational Tests, simulator studies, see [12]). The studies revealed several important results, e.g. that the hands-off mode does not lead to an impairment in visual attention compared to hands-on driving and that drivers adapt their level of motoric control to the situational circumstances. The slightly prolonged reaction time of 0.3 seconds to move the hands back to the steering wheel at functional limits in hands-off driving (also observed by [5,9,11]) can be compensated by a driver monitoring system assuring an adequate monitoring of the driving task. Furthermore, the authors of the report observed no increase in mode confusion if adequate information was provided about the drivers’ responsibilities and system functioning.

However, up to now, there are no studies available investigating the understanding of an L2 system including both, hands-off and hands-on function within one system (e.g. depending on the driven speed or the Operational Design Domain ODD). This combination could bring special demands on the understanding of the system and the potential danger that the hands-off system could be misinterpreted as a Level 3 system, in which the responsibility for the driving task can be completely handed over to the system for some time.

In addition, up to now the majority of the studies investigating L2 automation with hands-on and hands-off focus especially on the behavior at system limitations thus taking the functional design into account. In contrast, there is scarce research with a focus on the HMI itself and an accurate understanding thereof. This requires a different methodological approach with self-report data rather than behavioral data at system limits.

Finally, according to the studies conducted within the VDA project, there are no perceived and objectively measured safety impairments of hands-off systems in comparison to hands-on systems. Victor et al. [26] further observed that if drivers do not adequately monitor the driving situation and the system this is more a general problem of inadequate trust on the L2 system as one which is related to the fact of hands-off driving. The VDA report also provides design guidelines for L2 hands-off systems among about the necessary components that should be included, e.g. a Driver Monitoring System.

In hands-off systems, the requirement to stay attentive has to be ensured by other means than sensors in the steering wheel. Typically, the driver's visual attention behavior is tracked by a remote camera system in the vehicle interior that faces the driver and detects eye movements, head movements and/or body postures. If the system detects that the driver is not sufficiently attending the road and the environment but takes the eyes too long or too frequently to other areas of interest, a monitoring request (visual-auditory warning) is issued to redirect drivers’ visual attention towards the forward roadway. Up to now, only the Euro NCAP protocol Safety Assist [6] provides clear specifications on this attention monitoring and warning.

Several empirical studies investigated the potential of DMS systems considering driver's eyes-off behavior. The DMS used in Kurpiers et al. [14] consisted of a three-stage warning cascade with the first warning triggered after 4 seconds eyes-off road time. They reported improved monitoring behavior and an increase in trust and mode awareness as positive effects of the DMS. Blanco et al. [3] also showed that attention warnings were successful in getting participants to monitor the road whereas warnings after 2 s of visual inattention encouraged drivers to monitor the road more than warnings that are issued after 7 s. However, authors also revealed that attention reminders based on 2 s visual inattention lead to habituation effects and the participants start to ignore the prompts. Llaneras et al. [15] started a first DMS warning after 6 s and found improved reactions to silent malfunctions as a consequence. Victor et al. [26] reported a significant reduction of number of off-road glances that exceeded 2, 4, and 8 seconds when drivers received respective attention warnings triggered after these times. Bassani et al. [2] issued a first auditory warning (beep sound) after 2 s distracted driving (head and gaze not directly focused on the road) and 3 s later also a speech-based warning message. They reported an improvement in lateral control however a deterioration in speed maintenance.

As outlined above, there is already a body of research showing positive effects of DMS. However, all these studies were designed with a different methodological approach and thus lack comparability. As a consequence, it may be true that the system and HMI designs of the studies above provide positive results in the specific methodological context, but on the other side, it is not clear whether they may all be suitable solutions for a market ready product in a series production vehicle. Furthermore, it remains unclear how the combination of different L2 functionalities (e.g. hands-off driving mode and hands-on driving mode within one system) together with the respective DMS solutions affects the understanding of responsibilities of the drivers.

For this global evaluation of an L2 HMI it is necessary to have one common and standardized test protocol. However up to now, only few of such test protocols exist. One is described in Schömig et al., [22]; see also [23] for the German report]. This method was designed to capture a driver's interaction with a system via the HMI in specific scenarios in user studies. It evaluates several observable aspects of this interaction in real time and codes inadequate behavior in the categories “system operation”, “driving behavior” and “monitoring behavior”. A generic rating regarding the overall handling of the scenario is derived from these criteria. The test procedure includes a phase with detailed information about the system, its functionalities and potential limits but does not observe the intuitive interaction with the system. Thus, usability and intuitive usage are not captured by this protocol but rather safety of use over the long term. Such a focus requires observation of driving behavior and driver performance in safety-critical situations at system limits. Even though driver's subjective evaluations of his/her HMI understanding are included in the tool, it clearly focuses on the analysis of behavioral measures. The method for the evaluation of L2 HMI shifts this focus towards the assessment of the HMI's understandability by prioritizing self-reports of the drivers’ knowledge about the system states [1,10,16,24].

For the empirical evaluation of HMI of Automated Driving Systems (ADS, i.e. Level 3 and higher, a test protocol was defined by Naujoks et al. [20]. This test protocol aims to evaluate whether the basic HMI requirements as formulated by the NHTSA in their “Federal Automated Vehicles (AV) Policy” [19] are met by observing participants’ interactions with the HMI and self-reported understanding of the mode indicators presented during transitions and driving in the various system states. Specifics of the test protocol described in Naujoks et al. [20] concern the definition of a standardized use case set, an overview of evaluation criteria that should be used to decide whether the HMI is compliant with the basic HMI requirement and other methodological issues, e.g. the selection of an adequate study sample and the specification of instructions. The method for the evaluation of L2 HMI presented in this paper is based on this method.

## CRediT authorship contribution statement

**Nadja Schömig:** Conceptualization, Methodology, Investigation, Visualization, Project administration, Writing – original draft. **Christina Kremer:** Conceptualization, Methodology, Investigation. **Sebastian Gary:** Investigation. **Yannick Forster:** Conceptualization, Writing – review & editing, Supervision. **Frederik Naujoks:** Conceptualization, Supervision. **Andreas Keinath:** Funding acquisition. **Alexandra Neukum:** Funding acquisition.

## Declaration of competing interest

The authors declare that they have no known competing financial interests or personal relationships that could have appeared to influence the work reported in this paper.

## Figures and Tables

**Table A1:** Overview of possible use cases in drive A. A subset may be selected for a specific system to be tested.

Aut Level	UC No.	Use Case Name	Description
0–2	1	L0_L2_Activation	drive in L0; activation L2 (instructed)
2	2	L2_HandsOn_1	drive in L2 hands-on (1)
	3	L2_HandsOn_SetSpeed_1	automatic speed adaptation (1)
	4	L2_HandsOn_Off	transition hands-on → hands-off
	5	L2_HandsOff_1	drive in L2 hands-off (1)
	6	L2_degraded	failure in L2 lateral control hands-off
	7	L2_HandsOff_2	drive in L2 hands-off (2); lateral control active again
2–0	8	L2_L0_limit	system-initiated transition L2 hands-off → L0
0–2	9	L0_L2_standby	L2-activation (instructed)–lateral control in standby
2	10	L2_HandsOff_3	drive in L2 hands-off (3) lateral control available again
	11	L2_SetSpeed_degraded	automatic speed adaptation fails
	12	L2_L1_limit	system limit; transition L2 → L1
	13	L1_L2_Activation	reactivation L2 hands-off (instructed)
	14	L2_HandsOff_4	drive in L2 hands-off (4)
	15	L2_HandsOn_SetSpeed_2	automatic speed adaptation (2)
	16	L2_HandsOff_on	transition hands-off → hands-on
	17	L2_HandsOn_2	drive in L2 hands-on (2)
	18	L2_HandsOn_steer	steady state L2: driver steers (overrides lat. control)
	19	L2_HandsOn_accelerate	steady state L2: driver accel. (overrides long. control)
	20	L2_HandsOn_brake	steady state L2: driver brakes (deactivates system)
0–1-0	21	L0_L1_L0	driver-initiated activation and deactivation of L1

**Table A2:** Overview of possible use cases in drive B. A subset may be selected for a specific system to be tested.

Aut Level	UC No.	Use Case Name	Description/“instruction“
0	1	L0_L2_Activation	drive in L0; activation L2 (instructed) – “press button x”
2	2	L2_HandsOn	drive in L2 hands-on
	3	L2_HandsOn_SetSpeed	automatic speed adaptation works – “observe what system is doing“
	4	L2_SetSpeed_degraded	automatic speed adaptation fails – “observe what system is doing”
	5	HandsOff warnings	Driver receives HOW stages 1, 2 +3 stepwise – “please remove hands from the wheel” - “ignore first warning”—"ignore second warning”
	6	EyesOff warnings	Driver receives EOW stage 1+2 stepwise- “please look away from the road” – “ignore first warning”
	7	L2_HandsOn → L2 HandsOff	Triggered transition hands-on to hands-off; drive in hands-off
	8	L2_degraded	Triggered failure in L2 lateral control hands-off
1	9	L2_L1_limit	Triggered system limit; transition L2 → L1 - reactivation instructed
0	10	L2_L0_limit	Triggered system-initiated transition L2 hands-off → L0 – reactivation instructed
2	11	L0_L2_standby	Triggered non-availability of L2-lateral control- afterwards instruction: “press button x”
	12	L2_HandsOn_steer	Instruct driver to steer
	13	L2_HandsOn_accelerate	Instruct driver to accelerate
0	14	L2_HandsOn_brake	Instruct driver to brake

## Data Availability

Data will be made available on request.

## Appendix

Tables A1 and A2
